# Impact of *Wolbachia* on Infection with Chikungunya and Yellow Fever Viruses in the Mosquito Vector *Aedes aegypti*

**DOI:** 10.1371/journal.pntd.0001892

**Published:** 2012-11-01

**Authors:** Andrew F. van den Hurk, Sonja Hall-Mendelin, Alyssa T. Pyke, Francesca D. Frentiu, Kate McElroy, Andrew Day, Stephen Higgs, Scott L. O'Neill

**Affiliations:** 1 Public Health Virology, Communicable Diseases Unit, Queensland Health Forensic and Scientific Services, Coopers Plains, Australia; 2 School of Biological Sciences, Monash University, Clayton, Australia; 3 Institute for Health and Biomedical Innovation, Queensland University of Technology, Kelvin Grove, Australia; 4 Department of Pathology, University of Texas Medical Branch, Galveston, Texas, United States of America; USAMRIID, United States of America

## Abstract

Incidence of disease due to dengue (DENV), chikungunya (CHIKV) and yellow fever (YFV) viruses is increasing in many parts of the world. The viruses are primarily transmitted by *Aedes aegypti*, a highly domesticated mosquito species that is notoriously difficult to control. When transinfected into *Ae. aegypti*, the intracellular bacterium *Wolbachia* has recently been shown to inhibit replication of DENVs, CHIKV, malaria parasites and filarial nematodes, providing a potentially powerful biocontrol strategy for human pathogens. Because the extent of pathogen reduction can be influenced by the strain of bacterium, we examined whether the *w*Mel strain of *Wolbachia* influenced CHIKV and YFV infection in *Ae. aegypti*. Following exposure to viremic blood meals, CHIKV infection and dissemination rates were significantly reduced in mosquitoes with the *w*Mel strain of *Wolbachia* compared to *Wolbachia*-uninfected controls. However, similar rates of infection and dissemination were observed in *w*Mel infected and non-infected *Ae. aegypti* when intrathoracic inoculation was used to deliver virus. YFV infection, dissemination and replication were similar in *w*Mel-infected and control mosquitoes following intrathoracic inoculations. In contrast, mosquitoes with the *w*MelPop strain of *Wolbachia* showed at least a 10^4^ times reduction in YFV RNA copies compared to controls. The extent of reduction in virus infection depended on *Wolbachia* strain, titer and strain of the virus, and mode of exposure. Although originally proposed for dengue biocontrol, our results indicate a *Wolbachia*-based strategy also holds considerable promise for YFV and CHIKV suppression.

## Author Summary

Mosquito-transmitted viruses such as dengue, yellow fever and chikungunya, are responsible for significant morbidity and mortality throughout tropical and sub-tropical regions of the world. These viruses are primarily transmitted by *Aedes aegypti*, a mosquito that due to its close association with humans has historically been difficult to control. An innovative control strategy involving the release of mosquitoes infected with the intracellular bacterium *Wolbachia* is currently being developed. This approach is based on the recent discovery that *Wolbachia* reduces infection of mosquitoes with dengue virus, malaria parasites and filarial nematodes. In the current study, we demonstrated that *Wolbachia* also blocks infection of chikungunya and yellow fever viruses in *Ae. aegypti*. The degree of virus inhibition depended on the strain of *Wolbachia*, the route of virus exposure, the virus strain and the titer of virus that the mosquitoes were exposed to. The implementation of *Wolbachia*-based control strategies has the capacity to transform the way that mosquitotransmitted diseases are controlled in the future.

## Introduction

Mosquito-transmitted viruses cause significant human morbidity and mortality throughout the world and impose particularly heavy health and economic burdens on developing countries. Dengue, caused by infection with any of the four dengue virus (DENV) serotypes, is currently the leading arboviral disease, with millions of cases of classic dengue fever and tens of thousands of deaths annually due to hemorrhagic disease [1]. Yellow fever virus (YFV) has been implicated in an estimated 200,000 clinical cases and 30,000 human deaths annually in the equatorial regions of Africa and South America [2], [3]. Recently, chikungunya virus (CHIKV) emerged as a major threat, with unprecedented outbreaks on islands in the western Indian Ocean, as well as in India, Thailand, Malaysia and Italy [4]. Effective vaccines against all four DENV serotypes and CHIKV are still at various stages of development and clinical trial [5], [6]. Although a highly effective vaccine against YFV has been administered for over 50 years, rapid vaccination of susceptible populations either prior to or during an epidemic is financially and logistically challenging, particularly in developing countries [2], [3].

Current disease control measures focus on the suppression of mosquito vector populations to reduce virus transmission. The primary vector of DENVs, CHIKV and YFV is the mosquito *Aedes aegypti*, a highly domesticated species that feeds almost exclusively on humans. Its geographic range has expanded with increased urbanization, resulting in increased arbovirus transmission [7]. The primary mosquito control activities are source reduction to eliminate larval habitats, application of larvicides (such as Temephos or *s*-methoprene), or adulticiding with indoor residual spraying or ultra-low-volume application. While these approaches can be successful, they are often labor-intensive, can be prohibitively expensive to implement and require a sustained commitment from all levels of government [8]. Furthermore, increasing insecticide resistance and concerns with non-target effects on the environment have necessitated the development of alternative approaches to arbovirus control.

An emerging biocontrol approach to reducing transmission of arboviruses is provided by the transinfection of *Ae. aegypti* mosquitoes with *Wolbachia pipientis* from other insect hosts [9], [10]. *Wolbachia* is a maternally transmitted, endosymbiotic bacterium that manipulates host reproduction to enhance its own transmission [11]. Estimated to infect over 60% of insect species [12], *Wolbachia* can provide its host with nutritional benefits [13] and enhanced resistance to pathogens [14], [15]. *Ae. aegypti* transinfected with the *w*MelPop-CLA strain of *Wolbachia* from *Drosophila melanogaster* [16] displayed a shortened life span [16], a reduction in blood feeding success [17], [18] and dramatically lowered DENV serotype 2 (DENV-2) infection levels compared to *Wolbachia*-free control mosquitoes [19]. Although these phenotypes are likely to reduce virus transmission in the field, *w*MelPop-CLA may also impose fitness costs on *Ae. aegypti*, such as reduced fecundity due to poor blood feeding success [17], [18] and decreased embryonic viability [20].

A second strain of *Wolbachia*, *w*Mel that is closely related to *w*MelPop-CLA and also occurs naturally in *D. melanogaster* was recently introduced into *Ae. aegypti* as an additional strain for the biocontrol of dengue [21]. Both strains induce cytoplasmic incompatibility and have high rates of maternal transmission [16], [21], phenotypes necessary for the invasion of *Wolbachia* in natural populations of mosquitoes. Unlike *w*MelPop-CLA infected mosquitoes, *Ae. aegypti* infected with *w*Mel did not suffer any significant deleterious fitness costs when compared to uninfected controls [21]. Similar to *w*MelPop-infected *Ae. aegypti*, *w*Mel-infected mosquitoes displayed dramatically reduced replication of DENV-2 [21]. *Ae. aegypti* infected with *w*Mel were deployed in the field in north Queensland, Australia over the 2010–2011 summer [22]. *Wolbachia* was able to reach almost 100% fixation in wild mosquito populations only a few months following release, indicating that *Wolbachia*-infected mosquitoes present a very promising strategy for the control of dengue that is cost-effective and poses minimal environmental and social harm [23], [24].

Although originally developed as a biocontrol tool for DENVs, *Ae. aegypti* infected with *w*MelPop-CLA showed reduced infection with CHIKV [19], filarial nematodes [25] and avian malaria [19]. Therefore, *w*MelPop-CLA infected mosquitoes could potentially be used for biocontrol in areas where human pathogens other than DENVs occur. However, not all strains of *Wolbachia* protect equally well, with those phylogenetically most closely related to *w*Mel and *w*MelPop conferring the greatest degree of protection [26]. In *Drosophila*, *w*Mel confers protection against a range of viruses, for example Drosophila C virus, Flock House virus and Cricket paralysis virus [14], [15]. However, it remains unclear whether *w*Mel infection is able to limit the replication of human pathogens other than DENVs in mosquitoes, information that is critical to evaluating different *Wolbachia* strains for biocontrol.

Here, we tested the ability of the *w*Mel strain of *Wolbachia* to limit infection in *Ae. aegypti* with CHIKV and YFV. We also tested the ability of YFV to replicate in mosquitoes infected with *w*MelPop-CLA, in order to compare this strain to *w*Mel. We found reduced levels of CHIKV but not YFV infection in *w*Mel-infected *Ae. aegypti*, although the degree of virus inhibition depended on the mode of infection. By contrast, mosquitoes harboring the *w*MelPop-CLA strain of *Wolbachia* showed reduced infection rates with YFV with the extent of reduction virus strain and titer dependent.

## Materials and Methods

### Mosquitoes

Six different lines of *Ae. aegypti* were used in the experiments. The transinfection of *Ae. aegypti* with the *w*MelPop-CLA and *w*Mel strains of *Wolbachia* and maintenance of infected lines has been previously described 16,21. The *w*Mel-infected line, MGYP2, and its tetracycline-treated counterpart, MGYP2.tet, were exposed to both YFV and CHIKV. Both lines were in the F_16_ generation for the YFV experiments and the F_24_ generation for the CHIKV experiments. The original *w*MelPop-CLA infected line, PGYP1 [16], and the corresponding tetracycline-treated line, PGYP1.tet, were assessed in the YFV experiments only. The PGYP1 line was used in the combined F_52_ and F_53_ generations, while the PGYP1.tet was used in the combined F_50_ and F_52_ generations. A *Wolbachia*-uninfected wild-type line of *Ae. aegypti*, designated Cairns3, which originated from Cairns, Australia, was used as a positive control for all experiments. The YFV-susceptible Rex-D white-eye Higgs strain of *Ae. aegypti* that originated from Rexville, Puerto Rico, was used as an additional positive control for the YFV experiments [27], [28]. Both the Cairns3 and Rex-D lines had been in colony for >40 generations.

### Virus strains

The CHIKV strain was isolated from a patient visiting Melbourne, Australia in 2006 and contained the alanine to valine mutation in the membrane fusion glycoprotein E1 gene (E1-A226V) that has been linked to increased infectivity in mosquitoes, especially *Ae. albopictus* [29], [30]. The CHIKV stock had previously been passaged three times in African green monkey kidney (Vero) cells prior to use in this study. Two YFV strains that have been characterized in mosquitoes were used for the experiments: BA-55 which was isolated from a fatal yellow fever case in Nigeria in 1987 [31] and Cinetrop 28 (OBS 7549), which was isolated from a yellow fever patient in Bolivia in 1999 (R.B. Tesh, University of Texas Medical Branch, personal communication). The BA-55 strain had been passaged three times in suckling mouse brains, whilst the Cinetrop 28 strain had been passaged twice in C6/36 (*Ae. albopictus*) cells.

### Exposure of mosquitoes to YFV and CHIKV

The CHIKV experiments were undertaken in the Biosafety Level 3 (BSL-3) insectary at Queensland Health Forensic and Scientific Services, Brisbane, Australia, and the experiments with YFV were undertaken in the BSL-3 insectary located at the University of Texas Medical Branch, Galveston, Texas, USA. The MGYP2, MGYP2.tet and Cairns3 lines were exposed to CHIKV using both intrathoracic inoculation and oral feeding. Intrathoracic inoculation was employed because it circumvents the midgut infection and escape barriers, and allows a standard amount of virus to be delivered. The six *Ae. aegypti* lines were exposed to YFV via intrathoracic inoculation and in an infectious blood meal. However, low feeding rates in all lines coupled with unexpectedly low infection rates in control lines that did actually feed, compromised our ability to draw any meaningful conclusions from the YFV oral feeding data. Thus it was excluded from the current paper. For intrathoracic inoculation, immobilized mosquitoes were inoculated with 0.5 µl and 0.22 µl of YFV or CHIKV suspension, respectively. The suspension consisted of stock virus diluted in growth media (GM; Gibco-BRL, Gaithersburg, MD) which contained antibiotics and antimycotics and was supplemented with 10% fetal bovine serum (FBS) for YFV or 3% FBS for CHIKV. For oral feeding, mosquitoes were allowed to feed on a blood meal consisting of CHIKV diluted in commercially available defibrinated sheep blood (Institute of Medical and Veterinary Science, Adelaide, Australia) and 1% sucrose. The blood meal was housed within a membrane feeding apparatus that was fitted with pig intestine as the membrane [32]. Feeding was undertaken at 23°C and a 2 h period was used to ensure that a sufficient number of mosquitoes had imbibed a blood meal. Immediately following virus exposure, mosquitoes were anaesthetized with CO_2_ and engorged mosquitoes placed in 900 ml gauze-covered containers. All mosquitoes were maintained on 10% sucrose at 28°C, high relative humidity and 12L∶12D light cycle within an environmental growth cabinet.

### Infection and dissemination

For the YFV experiments, mosquitoes were processed at either day 10 or 14 post-exposure. Mosquitoes were chilled and the heads removed from the bodies and placed separately in 0.5 ml of GM supplemented with 10% FBS, and antibiotics and antimycotics. Recovery of virus from the head demonstrated that the virus had infected head tissue and was potentially able to be transmitted.

For the CHIKV experiments, infection, dissemination and transmission were assessed using a modified *in vitro* capillary tube system [33]. Briefly, mosquitoes were anaesthetized with CO_2_ and the legs and wings removed. The saliva was collected by inserting the proboscis of the mosquito into a capillary tube containing GM with 20% FBS. After 30 min, the contents of the capillary tube were expelled into 600 µl of GM with 3% FBS. The salivary glands were then dissected from the body in a drop of GM with 3% FBS. The dissected salivary glands were washed in a drop of 1% bleach [34], before being rinsed in 2 drops of GM with 3% FBS. The body remnants and salivary glands were placed in separate 2 ml tubes containing 1 ml of GM with 3% FBS and 3 sterile glass beads.

Salivary glands were also excised for visualization of CHIKV infection via an immunofluorescence assay (IFA). The glands were dissected in a drop of PBS and washed in another two drops of PBS, before each gland was placed in separate wells of an 18 well microscope slide. Slides were air-dried, fixed in cold acetone for 30 min, before being stored at −80°C.

### Virus assays

#### Titration of initial blood/virus mixtures

Blood/virus mixtures were titrated as serial ten-fold dilutions in 96 well microtiter plates seeded with confluent monolayers of C6/36 cells. Plates were incubated at 28°C for 5 days or 7 days for YFV- or CHIKV- infected blood/virus mixtures, respectively, before being fixed in PBS/acetone and stored at −20°C. Infection with YFV was detected using an indirect immunofluorescence assay with the monoclonal antibody (Mab) 863 [35]–[37], whilst CHIKV infection was detected using a cell culture enzyme immunoassay (CC-EIA; [38]) and the alphavirus-reactive Mab, B10 (provided by Roy A. Hall, University of Queensland, Brisbane, Australia).

#### Detection of YFV and CHIKV in mosquito components and saliva expectorates

Heads, salivary glands, bodies and body remnants were homogenized and clarified by centrifugation. The CC-EIA was used to detect CHIKV in all mosquito tissues and saliva expectorates. To further reduce bacterial and fungal contamination, the mosquito tissues and saliva expectorates were filtered through a 0.2 µm filter prior to being inoculated onto the C6/36 cell monolayers.

Salivary glands and saliva expectorates were also analyzed for CHIKV RNA using real time TaqMan RT-PCR. YFV viral RNA was detected in the heads and bodies using a YFV-specific TaqMan RT-PCR. Viral RNA was extracted from 140 µl of each of the mosquito components and the saliva expectorates using the QIAmp Viral RNA kit following the manufacturer's protocol (Qiagen, Clifton Hill, Australia), with modifications described in Moreira and others used for the YFV extractions [19]. The primer and probe sequences, preparation of synthetic controls, reaction mix components, and cycling conditions are the same as described previously [39], [40]. The CHIKV TaqMan RT-PCR was used to qualitatively detect viral RNA in the salivary glands and saliva samples, with a sample considered to be positive if the cycle threshold (*C*_t_) value was <40. The number of YFV RNA copies in heads and bodies was quantified using the TaqMan RT-PCR, with RNA standards produced for the relative quantification of YFV RNA copy numbers normalized to RNA levels of the *Ae. aegypti* house-keeping gene RpS17 [19].

#### IFA analysis of CHIKV in *Ae. aegypti* salivary glands

Fifteen microliters of B10 Mab was added to each well. After incubation at 37°C for 30 min in a humidified container, slides were rinsed in PBS pH 7.4 before being washed for 10 min in PBS pH 7.4. Slides were air-dried before the addition to each well of 15 µl Dako Anti-Mouse FITC Conjugate (Dako, Glostrup, Denmark) in 0.04% Evan's Blue in PBS pH 7.4. Slides were incubated and washed as described above, followed by application of two drops of Dako fluroescent mounting medium (containing NaN_3_; Dako, Glostrup, Denmark). A slide containing wells inoculated with Ross River virus, Barmah Forest virus, Sindbis virus and CHIKV infected cells was included as a positive control. Slides were examined under an Olympus CX41 fluorescent microscope (Olympus, Tokyo, Japan).

### Statistical analysis

Infection, dissemination and transmission rates were analyzed using Fisher's Exact Tests with two-tailed *P*-values. YFV copy numbers were tested for differences among mosquito lines using Wilcoxon rank sum tests for non-parametric data and *P*-values <0.05 were considered statistically significant. *P*-values were adjusted for multiple tests using a Bonferroni correction. All analyses were performed in R [41].

## Results

### *w*Mel inhibits infection and dissemination of CHIKV in *Ae. aegypti*

We challenged *w*Mel-infected (MGYP2 line) and uninfected mosquitoes (the tetracycline-treated MGYP2.tet and wild-type Cairns3 lines) with CHIKV in two replicate oral feeding experiments. In both experiments, fewer MGYP2 mosquitoes displayed CHIKV infection compared to MGYP2.tet and Cairns3 lines (**Table 1**). In experiment 1, virus was detected in significantly fewer MGYP2 (23%) mosquito bodies compared to MGYP2.tet (83%) and Cairns3 (58%) lines (*P*<0.01). Similarly, in experiment 2, CHIKV was detected in significantly fewer MGYP2 (30%) bodies compared to MGYP2.tet (70%) and Cairns3 (67%) lines (*P*<0.01).

**Table 1:** Effects of the *w*Mel strain of *Wolbachia* (MGYP2 line) on CHIKV infection and dissemination in *Ae. aegypti* 12 days after virus exposure.

		Body			Salivary glands			Saliva		
		% infected (N)			% infected (N)			% infected (N)		
Experiment	CHIKV titer (log_10_ TCID_50_/ml)	MGYP2	MGYP2.tet	Cairns3	MGYP2	MGYP2.tet	Cairns3	MGYP2	MGYP2.tet	Cairns3
1	7.3	23 (30)*	83 (24)*	58 (26)	33 (15)	66 (15)	60 (15)	3 (30)	17 (24)	8 (26)
		-	-	-	**20 (15)** *	**80 (15)** *	**66 (15)**	**10 (30)** *	**58 (24)** *	**35 (26)**
2	7.3	30 (30)*	70 (23)*	67 (30)	20 (15)	60 (15)	67 (15)	3 (30)	10 (23)	3 (30)
		-	-	-	**27 (15)**	**67 (15)**	**67 (15)**	**6 (30)** *	**47 (30)** *	**33 (30)**
3	4.5	86 (30)	100 (30)	100 (30)	73 (15)	100 (15)	100 (15)	10 (30)	20 (30)	27 (30)
		-	-	-	**86 (15)**	**100 (15)**	**100 (15)**	**36 (30)**	**63 (30)**	**60 (30)**
4	6.3	100 (30)	100 (30)	100 (30)	86 (15)	76 (14)	100 (15)	6 (30)	6 (30)	20 (30)
		-	-	-	**100 (15)**	**100 (14)**	**100 (15)**	**36 (30)**	**53 (30)**	**60 (30)**

Virus infection determined using CC-EIA and TaqMan RT-PCR assays (shown in bold), in mosquitoes exposed to CHIKV using oral feeding (experiments 1 and 2) and intrathoracic inoculations (experiments 3 and 4). The Body category refers to the infection rate in the body remnants that remained after the salivary glands were removed. *Wolbachia*-uninfected lines = MGYP2.tet and Cairns3. N = total number of mosquitoes tested.

* *P*<0.01 (Fisher's Exact Test, two-tailed *P*). For clarity, we only denote significant differences between MGYP2 and its tetracycline-treated counterpart.

Next, we used both CC-EIA and a CHIKV-specific TaqMan RT-PCR assay to track dissemination of the virus to the salivary glands and saliva following oral feeding experiments. CHIKV was detected in significantly fewer MGYP2 (20%) salivary glands compared to those from MGYP2.tet (80%) and Cairns3 (66%) lines (*P*<0.01) in experiment 1, but only when using the TaqMan RT-PCR assay (**Table 1**, in bold). The TaqMan RT-PCR assay detected more CHIKV-positive saliva expectorates than the CC-EIA (**Table 1**), suggesting that, if infectious particles were present in the saliva, they were below the threshold of CC-EIA detection. Nonetheless, in both oral feeding experiments CHIKV was detected in significantly fewer saliva expectorates in MGYP2 (10% and 6%) than MGYP2.tet (58% and 47%) (in both experiments *P*<0.01), but not Cairns3 mosquitoes.

We tested whether *w*Mel infection reduced CHIKV infection rates in *Ae. aegypti* using intrathoracic inoculations as the mode of virus delivery, in two separate experiments. Inoculations with CHIKV at titers of 10^4.5^ and 10^6.3^ TCID_50_/ml resulted in similar rates of virus infection among the three mosquito lines in bodies, salivary glands and saliva expectorates, using both CC-EIA and TaqMan RT-PCR for detection (**Table 1**, experiments 3 and 4). Rates of infection in bodies and salivary glands were consistently high across all three lines. A smaller percentage of saliva expectorates from MGYP2 mosquitoes were virus infected compared to MGYP2.tet and Cairns3 mosquitoes, but the difference was not statistically significant.

**Figure 1:**
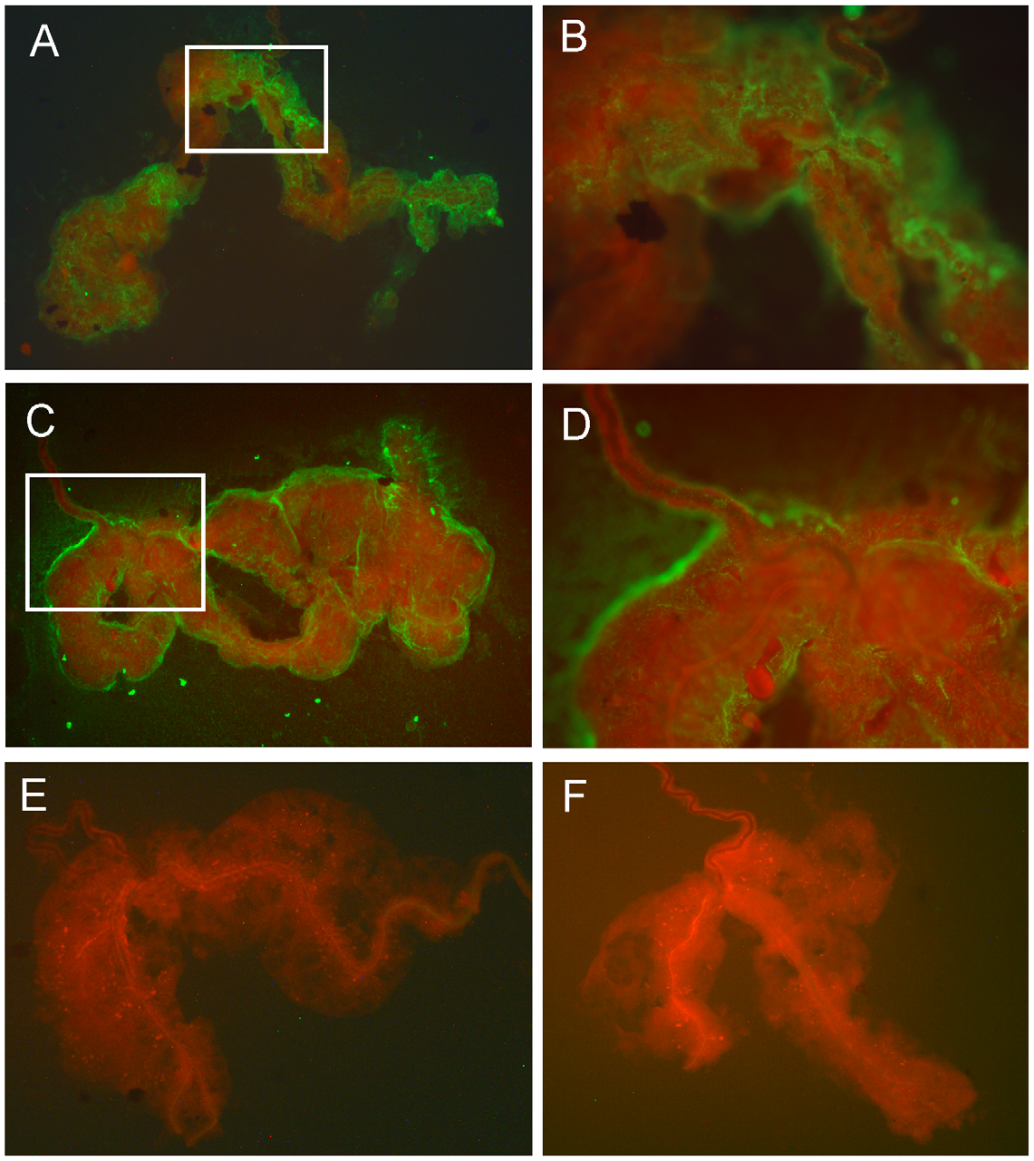
Chikungunya virus in the salivary glands of *Ae. aegypti*. CHIKV antigen (green fluorescence) detected in the salivary gland from a *w*Mel-infected mosquito (MGYP2) at 100× (**A**) and 400× (**B**) magnification, and from a *Wolbachia*-uninfected mosquito (Cairns3) at 100× (**C**) and 400× (**D**) magnification. Salivary glands negative for CHIKV antigen from MGYP2 (**E**) and Cairns3 (**F**) mosquitoes (Both glands are shown at 100× magnification).

Finally, we visualized CHIKV infection in the salivary glands of MGYP2 and Cairns3 *Ae. aegypti* lines using immunofluorescence assays (IFA). Twelve days following oral exposure, 27% (3/11) and 67% (6/9) of the MGYP2 and Cairns3 lines, respectively, were positive in the IFA. There did not appear to be any consistency in the localization of virus within infected salivary glands from either *Ae. aegypti* line (**Figure 1**).

### Reduced YFV infection rates in *w*MelPop-CLA but not *w*Mel-infected *Ae. aegypti*

We tested the effect of *w*Mel and *w*MelPop-CLA (PGYP1 mosquito line) *Wolbachia* strains on body infection rates, using intrathoracic inoculation with two strains of YFV isolated from Nigeria (BA-55) and Bolivia (Cinetrop 28). Four *Wolbachia*-uninfected mosquito lines were used as controls: Cairns3, Rex-D, MGYP2.tet and PGYP1.tet. Due to high mortality rates in some lines, mosquitoes exposed to 10^3.5^ TCID_50_/ml of YFV BA-55 were tested at 10 days post virus exposure. Mosquitoes exposed to10^4.5^ TCID_50_/ml of YFV BA-55 and 10^4.0^ TCID_50_/ml of YFV Cinetrop 28 were tested at 14 days post exposure.

At both low (10^3.5^ TCID_50_/ml) and high (10^4.5^ TCID_50_/ml) titers of YFV strain BA-55, MGYP2 mosquitoes had the same high (100%) rates of body infection as MGYP2.tet, Cairns3 and Rex-D mosquitoes (**Table 2**), detected using a YFV-specific Taqman assay. By contrast, at low titer, significantly fewer PGYP1 (12%) mosquitoes displayed body infections compared to the PGYP1.tet (100%), Cairns3 (100%) and Rex-D (100%) lines (*P*<0.001). PGYP1 mosquitoes also displayed significantly lower rates of body infection compared to MGYP2 mosquitoes (*P*<0.001). At the higher virus titer a similar number (100%) of PGYP1 mosquitoes was infected with YFV as for the PGYP1.tet, Cairns3 and Rex-D lines. A similar pattern was observed for the Cinetrop 28 strain of YFV, with mosquito body infection rates ranging from 96–100% and no significant differences among the mosquito lines, although this strain was tested at only one titer (10^4.0^ TCID_50_/ml).

**Table 2:** Effects of the *w*Mel and *w*MelPop-CLA (MGYP2 and PGYP1 lines, respectively) strains of *Wolbachia* on YFV infection and dissemination in *Ae. aegypti*.

		Mosquito lines					
YFV Strain	YFV titer (log_10_ TCID_50_/ml)	MGYP2	MGYP2.tet	PGYP1	PGYP1.tet	Cairns3	Rex-D
		% infected (N)	% infected (N)	% infected (N)	% infected (N)	% infected (N)	% infected (N)
BA-55	3.5	100 (13)*	100 (25)	12 (25)	100 (25)*	100 (25)*	100 (25)*
		**100 (13)** *	**100 (25)**	**0 (25)**	**100 (25)** *	**100 (25)** *	**92 (25)** *
BA-55	4.5	100 (34)	100 (25)	100 (18)	96 (23)	100 (16)	100 (25)
		**100 (34)**	**100 (25)**	**78 (18)**	**96 (23)**	**100 (16)**	**100 (25)**
Cinetrop	4.0	100 (31)	100 (15)	96 (25)	100 (25)	96 (24)	100 (25)
		**94 (31)**	**100 (15)**	**84 (25)**	**96 (25)**	**96 (24)**	**100 (24)**

Virus infection in bodies and heads (shown in bold) determined using the TaqMan RT-PCR assay. *Wolbachia*-uninfected lines = MGYP2.tet, PGYP1.tet, Cairns3 and Rex-D. N = total number of mosquitoes tested. Significant differences between PGYP1 and MGYP2, as well as PGYP1 and each of the control lines are denoted by

* *P*<0.001 (Fisher's Exact Test, two-tailed *P*).

Next, we assessed the impact of the two strains of *Wolbachia* on YFV dissemination to the head in *Ae. aegypti*. Using the YFV strain BA-55, MGYP2 mosquitoes displayed similar high rates of dissemination to the head as the MGYP2.tet, Cairns3 and Rex-D mosquitoes (92–100%), at both low and high virus titers (**Table 2**). At low virus titer, however, no dissemination to the head was observed in PGYP1 mosquitoes compared to control lines PGYP1.tet (100%), Cairns3 (100%), Rex-D (100%) and the MGYP2 (100%) (*P*<0.001). At high virus titer, a higher (78%) number of PGYP1 mosquitoes had virus in the heads and no significant differences in infection rates were found across the mosquito lines. High head dissemination rates (84–100%) were found across all mosquito lines challenged with the Cinetrop 28 strain of YFV, with no significant differences among them.

### Reduced YFV replication in *Wolbachia*-infected *Ae. aegypti* is virus titer and strain dependent

**Figure 2:**
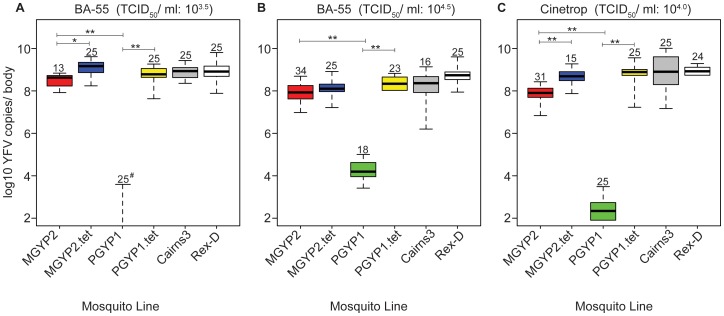
Effect of two *Wolbachia strains* on yellow fever virus replication in mosquito bodies. Viral RNA copies assayed by TaqMan RT-PCR in *w*Mel-infected (MGYP2), *w*MelPop-CLA infected (PGYP1) and *Wolbachia*-uninfected (MGYP2.tet, PGYP1.tet, Cairns3 and Rex-D) mosquitoes exposed to 10^3.5^ TCID_50_/ml (**A**) and 10^4.5^ TCID_50_/ml (**B**) of African (BA-55) and 10^4.0^ TCID_50_/ml (**C**) of South American (Cinetrop 28) YFV strains. Box plots show median RNA copy numbers and 25 (below median) and 75 (above median) percentiles, with outer bars at the lowest and highest observations (lower outer bars not shown when data overlaps zero). Number of mosquitoes assayed per line shown above box plots. #Denotes a median of zero. *P<0.01, **P<0.001 (Wilcoxon rank sum tests). For clarity, we only denote significant differences in comparisons between *Wolbachia*-infected lines and their corresponding tetracycline treated counterparts or between MGYP2 and PGYP1.

Next, we explored the effect of *Wolbachia* on the number of YFV RNA copies in *Ae. aegypti* bodies following intrathoracic inoculation with the strains BA-55 and Cinetrop 28, at three different titers. Overall, infection with both *Wolbachia* strains resulted in a lower median number of virus copies per body in *Ae. aegypti* compared to control lines. However, PGYP1 mosquito bodies consistently displayed several log fewer copies than MGYP2 lines (**Figure 2**). Significantly fewer copies were found in MGYP2 compared to *Wolbachia*-uninfected MGYP2.tet, Cairns3 and Rex-D mosquitoes (*P*<0.05 in all Wilcoxon rank sum tests) in challenges with YFV strain BA-55 at a titer of 10^3.5^ TCID_50_/ml. However, virus levels were high in both MGYP2 and the *Wolbachia*-uninfected lines (medians >10^8^ copies/body; **Figure 2A**). By contrast, median virus copy in PGYP1 mosquitoes was zero, an 8-log difference compared to MGYP2 (*P*<0.0001, Wilcoxon rank-sum test) and significantly less than PGYP1.tet, Cairns3 and Rex-D mosquitoes (*P*<0.0001 in all Wilcoxon rank-sum tests) (**Figure 2A**).

In challenges with YFV strain BA-55, at the higher titer of 10^4.5^ TCID_50_/ml, MGYP2 bodies still had significantly fewer virus copies than Rex-D (*P*<0.001, Wilcoxon rank-sum test) but not the MGYP2.tet or Cairns3 lines (**Figure 2B**). However, PGYP1 bodies had far less virus than either the MGYP2 or *Wolbachia*-uninfected lines (*P*<0.0001 in all Wilcoxon rank-sum tests) (**Figure 2B**). YFV replicated to a significantly lesser extent in MGYP2 and PGYP1 bodies following challenge with the Cinetrop 28 strain compared to control lines (*P*<0.0001 in all Wilcoxon rank-sum tests) (**Figure 2C**). YFV copy numbers were almost 5-logs lower in PGYP1 than MGYP2 bodies (*P*<0.0001, Wilcoxon rank-sum test) (**Figure 2C**).

**Figure 3:**
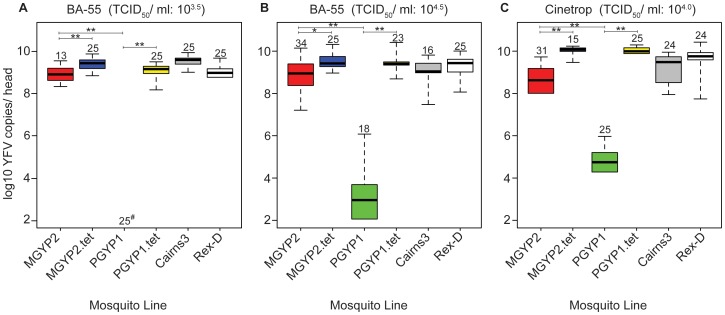
Effect of two *Wolbachia* strains on yellow fever virus replication in mosquito heads. Viral RNA copies assayed by TaqMan RT-PCR in *w*Mel-infected (MGYP2), *w*MelPop-CLA infected (PGYP1) and *Wolbachia*-uninfected (MGYP2.tet, PGYP1.tet, Cairns3 and Rex-D) mosquitoes exposed to 10^3.5^ TCID_50_/ml (**A**) and 10^4.5^ TCID_50_/ml (**B**) of African (BA-55) and 10^4.0^ TCID_50_/ml (**C**) of South American (Cinetrop 28) YFV strains. Box plots show median RNA copy numbers and 25 (below median) and 75 (above median) percentiles, with outer bars at the lowest and highest observations (lower outer bars not shown when data overlaps zero). Number of mosquitoes assayed per line shown above box plots. # Denotes a median of zero. *P<0.01, **P<0.001 (Wilcoxon rank sum tests). For clarity, we only denote significant differences in comparisons between *Wolbachia*-infected lines and their corresponding tetracycline treated counterparts or between MGYP2 and PGYP1.

We also explored whether *Wolbachia* strain and YFV inoculation titer influenced the extent to which the virus replicated in *Ae. aegypti* heads, using TaqMan RT-PCR quantification of viral RNA (**Figure 3**). In challenges with YFV strain BA-55 at the lower titer of 10^3.5^ TCID_50_/ml, significantly fewer RNA copies were detected in MGYP2 heads compared to *Wolbachia*-uninfected lines (*P*<0.001 in all Wilcoxon rank-sum tests), although the number of virus copies was still high (**Figure 3A**). By contrast, no virus was detected in the heads of PGYP1 mosquitoes. In challenges with YFV strain BA-55 at the higher titer of 10^4.5^ TCID_50_/ml, similar levels of virus disseminated to the head in MGYP2 as in Cairns3 and Rex-D, but not MGYP2.tet mosquitoes (*P*<0.01, Wilcoxon rank-sum tests) (Figure 3B). Median virus copy number for PGYP1 heads was six logs lower than the median for MGYP2 lines (*P*<0.0001, Wilcoxon rank-sum test), although virus was present in the majority of PGYP1 heads (**Figure 3B**). Significantly less virus had disseminated to MGYP2 and PGYP1 heads compared to control, *Wolbachia*-uninfected lines (*P*<0.0001 in all Wilcoxon rank-sum tests) when mosquitoes were challenged with the Cinetrop 28 strain (**Figure 3C**). Interestingly, a higher amount of YFV was found in PGYP1 heads in challenges with the Cinetrop 28 strain (median = 10^4.8^ copies, **Figure 3C**) compared to BA-55 at either low (median = 0 copies; **Figure 3A**) or high (median = 10^3.1^ copies; **Figure 3B**) inoculation titers, suggesting that the extent of YFV replication in the presence of *Wolbachia* may depend on virus strain.

## Discussion

Mosquito-transmitted viruses such as DENVs, CHIKV and YFV pose a significant health risk for almost half of the world's population. The release of *Wolbachia*-infected mosquitoes into natural populations has been proposed as a way of reducing DENV transmission while minimizing social and environmental harm [21], [22]. Although developed for dengue biocontrol, *Wolbachia*-infected mosquitoes may prevent the transmission of other significant arboviruses. Here, we evaluated whether mosquitoes infected with the *w*Mel strain of *Wolbachia* show limited infection with CHIKV and YFV. To compare the two *Wolbachia* strains, we tested whether YFV was able to infect and replicate in *w*MelPop-CLA infected mosquitoes.

Mosquitoes infected with *w*Mel showed significantly reduced rates of CHIKV infection and dissemination to the salivary glands compared to controls, but only in the oral exposure experiments. CHIKV also showed limited dissemination in *w*MelPop-CLA-infected mosquitoes following oral exposure [19], suggesting that both strains of *Wolbachia* may be useful candidates for release in CHIKV control programs. By contrast, YFV was much less likely to infect and disseminate in *Ae. aegypti* infected with *w*MelPop-CLA compared to *w*Mel strains. The virus was also less likely to replicate in *w*MelPop-CLA infected mosquitoes, with very high virus loads detected in *w*Mel-infected *Ae. aegypti*. Our experiments suggest that *w*MelPop-CLA infected mosquitoes may be the best candidates for YFV biocontrol programs, but were unable to determine the extent of virus replication following oral exposure rather than intrathoracic inoculation. Because virus inhibition with some *Wolbachia*-virus combinations does not appear to be complete, it is essential that epidemiological models be utilized to establish the threshold of virus inhibition necessary to minimize and prevent transmission in the field.

The *w*Mel strain occurs at high levels in *Ae. aegypti* ovaries and salivary glands but it is not present in as many body tissues as the *w*MelPop-CLA strain [19], [21]. In particular, *w*Mel does not accumulate to high levels in the fat bodies, brain and thoracic ganglia, which are involved in secondary replication of arboviruses prior to infection of the salivary glands [42]. YFV may have replicated to a sufficiently high level in these tissues in *w*Mel-infected mosquitoes to allow dissemination to the salivary glands and potentially be transmitted to humans. The presence of *w*Mel in the mosquito did not completely prevent the replication of CHIKV in *Ae. aegypti* salivary glands, as determined by cell-culture, real-time RT-PCR and IFA analysis.

Host immune pre-activation has been proposed as an explanation for interference with virus replication because *Wolbachia*-infected *Ae. aegypti* show significant immune upregulation [19], [25], [43]. Recently, Pan et al. showed that *Wolbachia* infection activates the Toll immune pathway in *Ae. aegypti* and the production of antimicrobial peptides which inhibit DENV-2 [44]. However, *Wolbachia* infection in *Drosophila*, the endosymbiont's native host, also blocks DENV-2 replication despite the absence of immune upregulation [45]. Therefore, although immune upregulation may explain some of the observed *Wolbachia*-mediated interference with virus replication, it may not be the whole explanation [45]. Alternatively, *Wolbachia* may compete with arboviruses for key cellular components [19], [46]. Fluorescence *in situ* hybridization has revealed that *w*MelPop-CLA infection is widespread throughout the body of infected *Ae. aegypti*, and is prevalent in tissues and organs associated with viral replication, including the fat bodies, brain and ommatidia [19]. Importantly, DENV-2 antigen did not co-localize in cells and tissues that were infected with *w*MelPop-CLA. As DENV-2 and YFV are closely related flaviviruses, it is possible that a similar pattern of virus exclusion is occurring with YFV in *w*MelPop-CLA infected cells and tissues. *In vitro* studies have shown that reduced virus replication correlates with the density of *Wolbachia* in the cell [46]. Further studies are required to determine the precise molecular mechanism underpinning *Wolbachia*-mediated interference with virus replication.

The discovery that *Wolbachia* limits arbovirus infection and replication in *Ae. aegypti* has the potential to fundamentally alter vector control strategies [23]. Importantly, mass release of *w*Mel-infected *Ae. aegypti* in two communities near Cairns, northern Australia, resulted in >90% frequency within the population six weeks after the first release [22]. Although the *Wolbachia*-based control strategy was initially proposed to control DENVs, previous work and the current study have demonstrated that releases of *Wolbachia*-infected *Ae. aegypti* to control DENVs may have the added benefit of reducing CHIKV and YFV transmission. Alternatively, *Wolbachia*-infected *Ae. aegypti* could be released to specifically reduce transmission of these viruses, irrespective of whether DENVs were circulating or not in an area.

We have demonstrated that the level of virus blockage induced by *Wolbachia* is dependent on the strain of *Wolbachia*, the strain of virus and the mode of exposure to the virus. Higher YFV intrathoracic inoculation titers allowed more breakthrough of virus in *w*MelPop-CLA infected mosquitoes. Infection rates, accumulation and dissemination to the head also differed between BA-55 and Cinetrop 28 in the presence of wMelPop-CLA, suggesting that different virus strains may have different replication dynamics in *Wolbachia*-infected mosquitoes. The CHIKV experiments showed that the TaqMan RT-PCR was generally more sensitive than the CC-EIA in detecting the presence of virus. This was not unexpected, given that the TaqMan RT-PCR does not differentiate between RNA derived from live, packaged infectious virus particles, from that of dead or defective, non-infectious virus. Thus, our experiments illustrate the importance of carefully selecting virus titer, virus strain, mode of delivery and method of virus detection when assessing the potential utility of different *Wolbachia* strains for biocontrol.

As part of the implementation of *Wolbachia*-based control strategies, it will be essential to assess the virus blocking ability of different *Wolbachia* strains against different virus strains, and if necessary, identify additional *Wolbachia* strains that could be deployed in the field. Commensurate with this will be the need for ongoing monitoring of *Wolbachia*-infected *Ae. aegypti* populations to confirm that the virus-limiting phenotype is maintained in successive generations post release.
